# Association of Genetic Risk for Age-Related Macular Degeneration with Morphological Features of the Retinal Microvascular Network

**DOI:** 10.3390/diagnostics14070770

**Published:** 2024-04-05

**Authors:** Adam Sendecki, Daniel Ledwoń, Aleksandra Tuszy, Julia Nycz, Anna Wąsowska, Anna Boguszewska-Chachulska, Adam Wylęgała, Andrzej W. Mitas, Edward Wylęgała, Sławomir Teper

**Affiliations:** 1 Chair and Clinical Department of Ophthalmology, Faculty of Medical Sciences in Zabrze, Medical University of Silesia, 40-752 Katowice, Poland; adam.sendecki@gmail.com (A.S.); aniadok@genomed.pl (A.W.); awylegala@sum.edu.pl (A.W.); ewylegala@sum.edu.pl (E.W.); slawomir.teper@sum.edu.pl (S.T.); 2 Faculty of Biomedical Engineering, Silesian University of Technology, 41-800 Zabrze, Poland; aleksandra.tuszy@polsl.pl (A.T.); andrzej.mitas@polsl.pl (A.W.M.); 3 Institute of Biomedical Engineering and Informatics, Technische Universität Ilmenau, 98693 Ilmenau, Germany; julia.nycz@tu-ilmenau.de; 4 Genomed S.A., 02-971 Warszawa, Poland; annab-ch@genomed.pl

**Keywords:** age-related macular degeneration, polygenic risk score, optical coherence tomography angiography, retinal image analysis, retinal vascular network

## Abstract

Background: Age-related macular degeneration (AMD) is a multifactorial disease encompassing a complex interaction between aging, environmental risk factors, and genetic susceptibility. The study aimed to determine whether there is a relationship between the polygenic risk score (PRS) in patients with AMD and the characteristics of the retinal vascular network visualized by optical coherence tomography angiography (OCTA). Methods: 235 patients with AMD and 97 healthy controls were included. We used data from a previous AMD PRS study with the same group. The vascular features from different retina layers were compared between the control group and the patients with AMD. The association between features and PRS was then analyzed using univariate and multivariate approaches. Results: Significant differences between the control group and AMD patients were found in the vessel diameter distribution (variance: *p* = 0.0193, skewness: *p* = 0.0457) and fractal dimension distribution (mean: *p* = 0.0024, variance: *p* = 0.0123). Both univariate and multivariate analyses showed no direct and significant association between the characteristics of the vascular network and AMD PRS. Conclusions: The vascular features of the retina do not constitute a biomarker of the risk of AMD. We have not identified a genotype–phenotype relationship, and the expression of AMD-related genes is perhaps not associated with the characteristics of the retinal vascular network.

## 1. Introduction

Age-related macular degeneration (AMD) is one of the leading causes of legal blindness in the industrialized world. AMD is characterized by the accumulation of extracellular deposits, namely drusen, along with the progressive degeneration of photoreceptors and adjacent tissues. AMD is a multifactorial disease that encompasses a complex interplay between aging, environmental risk factors, and genetic susceptibility. Chronic inflammation, lipid deposition, oxidative stress, and impaired maintenance of the extracellular matrix are strongly involved in the pathogenesis of AMD [1,2,3,4,5,6].

The retinal pigment epithelium (RPE) plays a crucial role in the degradation of photoreceptor cells in the retina. The RPE engulfs and breaks down the photoreceptor cells through phagocytosis, converting them into lipofuscin within its lysosomes. However, over time, lipofuscin accumulation in the RPE cells can lead to toxic reactions and, eventually, cell death [7].

Several genetic factors, lipid metabolism, oxidative stress, and aging are involved in the development of AMD. Dysregulation of genetics accounts for 46–71% of disease contribution, with CFH and ARMS2/HTRA1 identified as the two most significant risk loci among the 103 AMD-associated loci identified to date [8]. The genetic contribution to the development of AMD has been determined through linkage studies and genome-wide association approaches, which have revealed risk loci for AMD and several candidate genes. These include CFH, C3, C2-CFB, CFI, and a region on chromosome 10 that includes HTRA1/LOC387715/ARMS2, CETP, LIPC, VEGF-A, and APOE [9]. Although the exact pathophysiological mechanisms underlying AMD are still being investigated, there is general agreement that AMD is a multifactorial disease resulting from the synergistic effect of modifiable factors (e.g., smoking, eating habits, physical activity) and non-modifiable factors (age and genotype) [10]. The polygenic risk score (PRS) is used in numerous studies and is intended to estimate the risk of disease in various populations [11,12,13,14,15].

A key factor in the resulting retinal degeneration is reactive oxygen species (ROS) production. Increased levels of reactive oxygen species lead to damaged mitochondrial DNA (mtDNA), resulting in dysfunctional mitochondria, and, consequently, mtDNA causes further damage to retinal tissue [3]. These ROS are generated mainly when RPE cells are exposed to blue light within the range of 390 to 550 nanometers. This exposure triggers degenerative and apoptotic reactions in various retinal cells, including RPE cells, photoreceptors, pericytes, and endothelial cells [16,17].

The occurrence of oxidative stress triggers a cascade of events, including inflammation, which subsequently releases angiogenic factors [2]. In ocular diseases, neovascularization can be triggered by abnormal conditions such as ischemia, hypoxia, inflammation, and genetic predisposition. The presence of these conditions can lead to the stimulation of new blood vessel growth in the retina or choroid as a response to reduced oxygen supply to the affected area [18]. Neovascular age-related macular degeneration (nvAMD) is a condition that requires frequent and long-term intravitreal injections to preserve vision with a significant treatment burden to maintain sight [19,20,21].

nvAMD is a multifactorial disease with numerous risk factors that are thought to play a role in its development. Non-modifiable risk factors include increased age, female sex, genetic factors, Caucasian race, and light iris color. Modifiable risk factors include smoking, increased BMI, alcohol intake, and dietary habits [4]. Anti-VEGF therapies have emerged and are widely used, such as ranibizumab (Lucentis^®^, Novartis, Basel, Switzerland), aflibercept (Eylea^®^, Bayer, Berlin, Germany), brolucizumab (Beovu^®^, Novartis, Basel, Switzerland), faricimab (Vabysmo^®^, Roche, Basel, Switzerland), and the off-label use of bevacizumab (Avastin^®^, Roche, Basel, Switzerland) [21,22].

As the population continues to age, the incidence cases of AMD in general and neovascular AMD are projected to increase [5]. A study by Johanna M. Colijn et al. predicts that, by 2040, the number of individuals in Europe with early AMD will range between 14.9 and 21.5 million, and for late AMD, between 3.9 and 4.8 million [23].

Angio OCT, also known as optical coherence tomography angiography (OCTA), is a non-invasive imaging technique that allows the visualization of the microvasculature of the retina and choroid. It provides detailed information about blood flow in these structures without injecting any contrast agents [17]. Angio OCT utilizes the principles of OCT imaging based on low-coherence interferometry. It measures the interference pattern of light waves reflected from different depths within the tissue to create high-resolution cross-sectional images [24]. By detecting the dynamic movement of red blood cells, OCTA provides a unique perspective on blood flow in the retina and choroid. Angio OCT extends the capabilities of traditional OCT by incorporating motion contrast imaging. This innovative imaging technique enables a comprehensive analysis and visualization of the vascular structure of macular neovascularization (MNV), offering valuable insights into both physiological and pathological blood flow [25].

The retinal superficial capillary plexus (SCP) comprises the top three layers, housing arteries and veins responsible for the blood circulation in the deeper retina. Below these superficial layers, the deep capillary plexus (DCP) contains intermediate and deep capillaries. Notably, the SCP features larger, more prominently visible blood vessels due to its proximity to the imaging source, while the DCP displays finer vessels [24]. The choriocapillaris forms a densely interconnected network of capillaries, and a healthy choriocapillaris presents a distinct grainy texture in OCTA images. In contrast, the choroidal layer contains larger vessels in comparison to the retinal vasculature. Nevertheless, these larger choroidal vessels are not as well visualized in OCTA images, due to factors such as imaging depth limitations, signal attenuation, shadowing effects, the high reflectivity of the layers above, and the specific characteristics of blood flow [26,27].

The aim of the present study is to analyze the association between the characteristics of the retinal vascular network and the estimated risk of AMD based on genotypic analysis expressed as a PRS. To our knowledge, no articles have been published so far on OCTA images and the PRS to assess the relations between the vascular pattern in the macula and its deviations from the norm and genetic risk of AMD.

## 2. Materials and Methods

### 2.1. Dataset

Participants in the study were recruited by the Chair and Clinical Department of Ophthalmology at the Faculty of Medical Sciences in Zabrze, Medical University of Silesia in Katowice. The study was conducted in accordance with ethical guidelines and received approval from the Ethics Committee of the Medical University of Silesia (Resolutions No KNW/0022/KB1/105/13 and BNW/NWN/0052/KB1/97/I/22/23), following the principles outlined in The Declaration of Helsinki. Written informed consent was obtained from all participants after being informed of the purpose and protocol of the study.

The cohort comprised 332 patients, including 235 diagnosed with AMD and 97 healthy controls without symptoms of retinal degeneration. Individuals over 50 years of age were invited to participate, with AMD patients recruited during detailed follow-up visits at the ophthalmology clinic, including advanced forms of degeneration (neovascular AMD, geographic atrophy, and subretinal fibrosis) and early and intermediate forms. The control group included individuals recruited during follow-up visits after cataract surgery or planned check-ups, where fundus examination did not reveal any signs of AMD. The cohort included 20 eyes of patients treated for glaucoma (intraocular pressure was well controlled on one type of antiglaucoma drops): 8 of them were in the control group, and 12 were in the study group. The treatment received was similar in both groups.

Exclusion criteria for the study encompassed patients with macular diseases other than AMD (e.g., diabetic macular edema, macular dystrophy), limited vision that hinders fundus evaluation, prior retinal or choroidal inflammatory diseases, retinal detachment, intraocular procedures other than cataract surgery or posterior capsulotomy, and retinal laser therapy.

The study involved various ophthalmological examinations, such as best-corrected visual acuity (BCVA) using ETDRS charts, anterior segment examination with slit lamp biomicroscopy, and pupillary dilation with 1% tropicamide. A clinical examination of each eye using Volk superfield aspheric lenses 90D was performed to assess the posterior segments and further diagnostics to identify pathological changes in the macula. Digital fundus images, swept-source optical coherence tomography (SS-OCT) of the macula (radial and 3D wide scanning protocols), and optical coherence tomography angiography (OCTA) were obtained using the DRI OCT Triton tomograph from Topcon Healthcare, Tokyo, Japan. In this study, we used macular 6 mm × 6 mm blood flow OCTA images. Four en-face reconstructions were made based on segmentations of retinal layers in ranges defining the superficial capillary plexus (SCP), deep capillary plexus (DCP), outer retina (OR), and choriocapillaris (CC).

### 2.2. PRS Calculation

Ulańczyk et al. conducted research on 30 genes associated with critical retinal functions, including regulation of inflammation (e.g., TGFB1), immune response (e.g., C2, C3, CFB, CFH), lipid and protein synthesis (e.g., ELOVL4, HTRA1, RPL1) and the structure of the retinal layer (e.g., BEST1, C1QTNF5, GUCA1B). Other genes impact oxidative stress, extracellular matrix maintenance, transmembrane transport, transcription regulation, DNA repair, and AMD (e.g., ARMS2). Molecular Inverted Probes were used to enrich coding regions with flanking sequences. Subsequently, libraries were sequenced using the Illumina platform. The targeted enrichment of coding sequences from the 30 AMD-associated genes was achieved through this approach. The entire process took place at Genomed S.A., Warsaw [14].

The bioinformatic analysis included adapter trimming using Cutadapt v1.14 [28], reads mapping to the GRCh37.13 reference genome with Burrows–Wheeler Aligner v0.7.10, and deduplication based on Unique Molecular Identifiers using in-house scripts. For indel realignment and base recalibration, researchers used Genome Analysis Toolkit v3.5 (GATK) [29], a widely recognized and powerful software tool for best practices in these specific areas, ensuring the highest quality and accuracy of the results obtained.

The variant calling utilized HaplotypeCaller and UnifiedGenotyper from the GATK package to optimize single nucleotide variant and indel identification. Criteria included excluding variants missing in <95% of samples for data completeness and filtering out low-coverage variants (<10× in 80% of genotypes) to enhance reliability and accuracy.

In this study, data from a subgroup of subjects, previously considered by Wąsowska et al., were used in the PRS calculation using PLINK for QC of genotyped data and the additive model available via PRSice2 software (version 2.3.5). The PRS study was performed on the Polish population based on the targeted sequencing data involving the enrichment of genes known to be associated with AMD at the time of the study [30].

### 2.3. OCTA Image Selection

**Figure 1:**
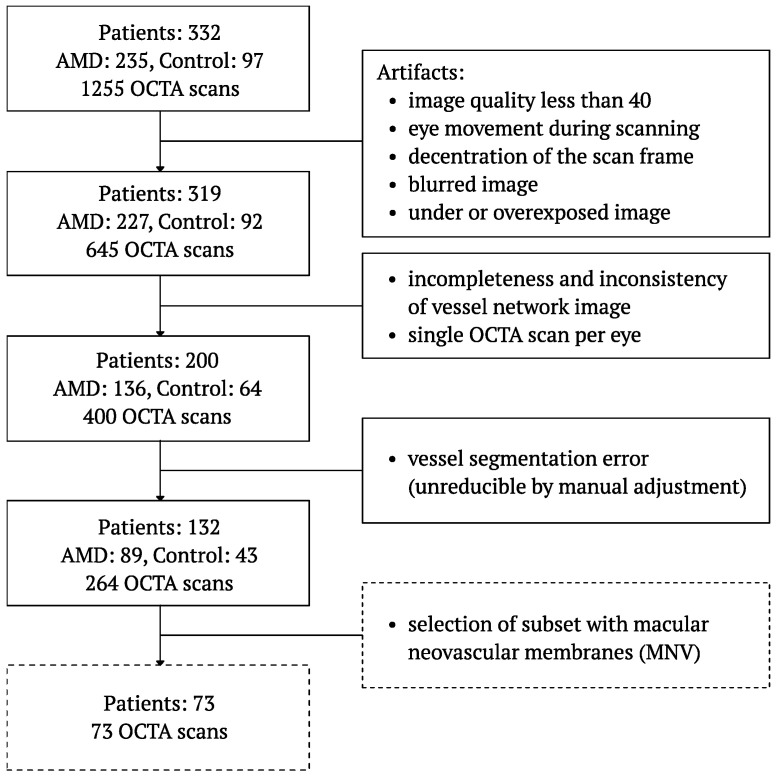
Flow chart of OCTA image selection into subgroups considered in the study.

Figure 1 shows the flow chart of OCTA image selection based on objective exclusion criteria and experts’ evaluation. The best-quality images were selected from a database of 1255 reconstructed OCTA reports. Images with artifacts that distorted the vascular pattern in the OCTA image or showed poor scan quality were rejected. We avoided using images with poor resolution, high levels of noise, or significant artifacts that can detract from the clarity and interpretability of the content. Over-processing was also taken into consideration; it was important to avoid excessive image processing that may lead to the introduction of artifacts, loss of detail, or misrepresentation of the original data. Image processing was abandoned due to its substantial influence on quantitative features of the vessels’ network morphology.

Motion or doubling artifacts, blurry images, quality scores less than 40, or poor centration were the first stage exclusion criteria for the analysis. The following stages of image rejection were carried out based on the results of the segmentation algorithm, using a possible change in algorithm parameters to improve the results. Moreover, only those patients for whom correct OCTA images were found for the right and left eyes were included in further analysis. As a result of the selection, the accurate OCTA images of 132 patients were selected, allowing proper segmentation of the vessels in the SCP layer. In addition, a subgroup of 73 patients with nvAMD in whom MNV was evident on CC or OR layers of OCTA images was selected for detailed analysis.

### 2.4. Feature Extraction

Quantitative evaluation of the OCTA images obtained was performed based on automated analysis using OCTAVA (OCTA Vascular Analyzer) software (version 1.0) developed by Untrach et al. [31] and semi-automatic analysis in IMAGEnet^®^ 6 software (version 1.26.16898, Topcon, Tokyo, Japan). The SCP images were preprocessed for noise detuning, shadow elimination, and enhancement of pixel position information using rolling ball background subtraction [32]. The processed images were then segmented and skeletonized in OCTAVA software. Vessel segmentation was carried out using the fuzzy means method and Frangi filtering with the maximum kernel value set to 2. After automatic skeletonization, the software assigns vessels and their connections to different categories. On the basis of this, quantitative indices of vessel area and length density (VAD and VLD, respectively), total number of nodes, branchpoint density (BD), fractal dimension (FD), and length, diameter, and tortuosity distributions are then calculated. Each feature describes a different aspect of morphology of the retina microvascular network. Feature distributions in a single image are presented in the form of descriptive statistics: mean, median, variance, skewness (measure of the asymmetry), and kurtosis (measure of the peakedness or flatness of the distribution). Based on the contours marked manually in IMAGEnet^®^ the foveal avascular zone (FAZ) areas in the SCP and DCP layers were calculated. For a selected subset of MNV, the contour of neovascularization was marked in the CC and OR layers, for which the area was also calculated.

### 2.5. Statistical Analysis

The determined variables were used as a first step in a comparative analysis between control subjects and AMD patients. For this purpose, the t-test was used if the assumptions of normality of distributions and homogeneity of variance were met (Shapiro–Wilk and F tests, respectively). Otherwise, the Mann–Whitney U test was used. The values for both eyes of each subject were averaged beforehand so that one subject represented a single observation. Univariate testing of the correlation of individual vessel morphological features with PRS was performed for the averaged values using the Pearson correlation coefficient. The general association of features with PRS values in the multivariate approach was verified using the generalized estimating equation (GEE) linear model. This approach allows the features of each eye to be used independently through the ability to model repeated measures. The influence of covariates in the form of subjects’ age at examination, gender, pigment epithelial detachment (PED), MNV, and AMD were included in this analysis. The significance level was established at 0.05.

## 3. Results

**Figure 2:**
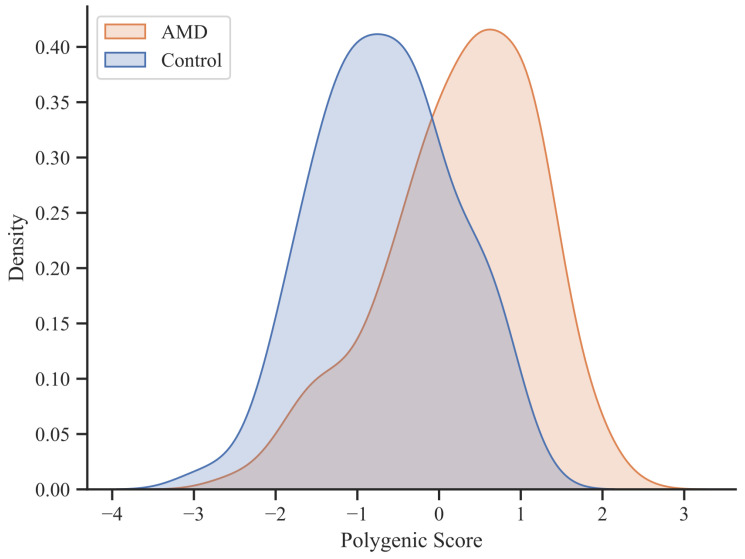
Distribution of PRS values per individual as density plot.

Figure 2 shows the distributions of PRS values that represent the differences between the AMD patient group and the control group resulting from the first part of the ongoing study, based on the total data collection. The statistical analysis revealed a significant difference in PRS between groups ($p=4.93\times 10^{-15}$) with a large effect size (r=0.58).

Table 1 presents the characteristics of the control group and patients with AMD from the selected dataset. There were no significant age differences between AMD patients and the control group. Excluding part of the data due to the established criteria for the OCTA SCP image introduced significant differences in the sex ratio between the groups. As expected, there was a significant deterioration in visual acuity and a reduction in choroidal thickness in the AMD group relative to the control group.

**Figure 3:**
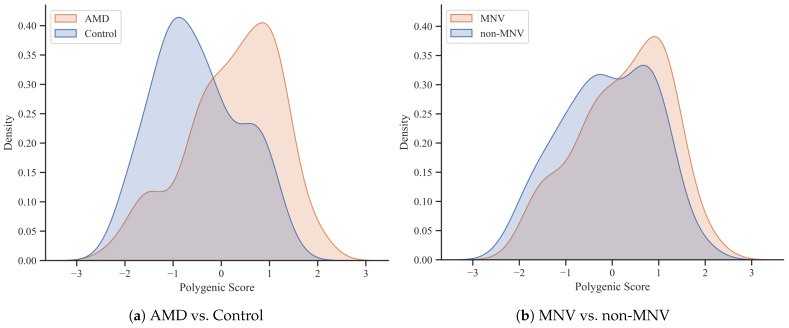
Distribution of PRS values for AMD patients and control group (**a**) and for MNV and non-MNV subjects (**b**) from the subset of participants with proper OCTA images selected.

Image selection reduced the number of study participants and, consequently, the number of unique PRS values for individual subjects. Despite this, the significant difference of PRS between groups has been preserved ($p=1.02\times 10^{-8}$, r=0.43) (Figure 3a). In the subgroup of subjects diagnosed with MNV based on OCTA analysis, the PRS value is significantly higher than the group without MNV (p=0.013, r=0.19). However, this difference is much smaller and may result from the disproportion in numbers between the AMD and control groups. Moreover, the range of PRS values in the MNV group is relatively wide and includes both subjects with low and high risk of AMD (Figure 3b). The results confirm the feasibility of analyzing the association of OCTA characteristics with PRS in the defined groups.

**Figure 4:**
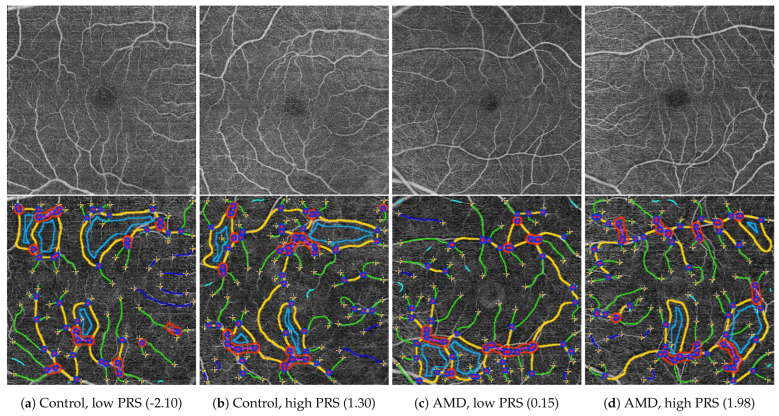
Example of en-face OCTA images (**top row**) and corresponding classification results of different architectural components of the vessel network (**bottom row**) for subjects from the control group and with AMD, for different AMD PRS values.

Figure 4 shows sample OCTA images from the dataset after selection according to established criteria, based on visual assessment. The presented results of OCTAVA software on images after preprocessing demonstrate the possibility of its effective use in the quantitative analysis of morphological features of the retinal microvascular network. The graphical presentation of the classification of individual vessels and structures into subclasses was the basis for the subsequent stages of selection of the analyzed dataset. The values of individual morphological features of the retinal microvascular network were determined based on the results of the classification.

The results of the statistical analysis of the difference in feature values between the group of patients with AMD and the control group are shown in Table 2. The mean FD is significantly larger in the subjects with AMD. The higher the FD, the more complex and developed the structure of the blood vessels is; we find the FD parameter as a general measure of the complexity of the blood vessel network. The standard deviation of FD is significantly higher in AMD. This indicates a greater variability of the complexity of the vessel structure within one image. The standard deviation of the vessels’ diameter is greater in AMD. Furthermore, another characteristic that differs significantly in patients with AMD is the skewness of the diameter distribution of the vessel. In both groups, it is negative, which means that there are more vessels in the image with a much smaller diameter compared to the diameter of the remaining vessels (many outliers with low values). In AMD, the skewness was greater (closer to zero), which means that fewer very thin vessels (much thinner than the rest) appeared in the images of AMD vessels than in the control group. In both cases, the skewness is greater than −1, which means that the distribution is close to normal, so the number of outliers is small.

Table 3 shows the results of the univariate and multivariate investigation of the relationship between individual morphological features of the retinal microvascular network and PRS including additional covariants. Using both correlation analysis and the GEE linear regression model, neither showed a direct and significant association of the characteristics of the vascular network with AMD PRS.

The general observation is that there is no association between the vascular network and PRS. The differences between FD and diameter characteristics in AMD and the control group are secondary to the occurrence of AMD. They do not constitute a biomarker of the risk of AMD. There is no genotype–phenotype relationship and the expression of AMD-related SNPs is not related to the characteristics of the retinal vascular network. It is difficult to clearly say whether these vessels are difficult to detect or whether they are absent. This may be due to retinal architecture disorders secondary to changes at the border of the RPE and outer retina.

## 4. Discussion

OCTA is a test whose interpretation may be problematic due to numerous artifacts occurring in the results. The rather long scanning time and difficulties in cooperation with the patient may pose difficulties. Problems with cooperation during examinations and eye movement artifacts during long examination time (longer than 10 s per eye) led to the rejection of many scans during our analysis. OCTA is a diagnostic tool that still requires the development of appropriate algorithms that integrate individual B-scans into a whole image [18]. Artifacts were also a problem with the segmentation algorithm, which sometimes marked an obvious line of artifact as a vessel. Rejecting such photos also narrowed the number of studies eligible for further analysis. The imperfection of this vascular imaging method was also highlighted in our study, where we struggled with many artifacts in the raw OCTA data. In three selection stages, we selected high-quality images for which the correct automatic segmentation of vessels was possible. The distribution of PRS values in the selected dataset correctly reflects the distribution in the entire dataset. However, the reduction resulted in a difference in the gender ratio between the group of patients with AMD and the control group. In the group of patients with AMD, the percentage of women was 55.05%, and the rate of men was 44.95%, while in the control group, a larger percentage was women (76.75%) and a smaller percentage was men (23.25%).

During the study, researchers repeatedly reported the need to manually mark the area of neovascularization and FAZ due to the imperfection of automatic algorithms [27]. When working with OCTA, it is important to consider the impact of low signal strength on image quality. Manufacturers recommend specific thresholds for sufficient signal strength, and, even if these measures are met, signal strength can still affect quantitative results [33,34]. For instance, improving signal strength may lead to an increase in retinal vessel densities. Therefore, ensuring adequate signal strength is crucial for accurate OCTA assessments [33,35]. In addition to signal strength, the positioning of scanning frames according to anatomical landmarks plays a significant role in ensuring comparability and reproducibility of quantitative OCTA images. Scanning frames are typically centered on the optic nerve head or the fovea. However, it is important to be aware of centration artifacts that can occur during OCTA analysis, with their frequency varying depending on whether the scanning frames are positioned manually or automatically by the OCTA device [18]. These artifacts are less relevant for smaller areas of interest, such as the foveal avascular zone (3 mm × 3 mm scans), but become critical when evaluating larger areas like the whole perifoveal macula (9 mm × 9 mm or 12 mm × 12 mm scans). The study conducted by Wicklein et al. has emphasized the importance of confirming accurate centration in every OCTA image to account for potential technical impacts on quantitative measures [36]. During our study, a panel of experts verified the correctness of the central positioning of the scanning frame in relation to the avascular zone or central neovascularization.

Furthermore, algorithmic failure in the segmentation of different retinal layers, such as the inner limiting membrane, the inner plexiform layer, or the retinal pigment epithelium, can lead to inaccurate mapping of blood flow signals to the retinal vascular plexus [3]. This issue is especially relevant for OCTA images incorporating retinal pathologies, as disruptions of the retinal architecture can severely affect segmentation and image quality [3,37]. It is essential to conduct a thorough assessment of accurate retinal layer segmentation within different B-scans to ensure correct segmentation, considering the potential impact of retinal pathologies on both retinal layer architecture and vasculature when interpreting OCTA images [38]. In our study, we also noticed similar problems: the proximity of superficial and deep plexuses and choriocapillaris, as well as their different thicknesses in different retinas, resulted in the interpenetration of these images, which led to incorrect automatic segmentation and necessitated the intervention of experts to correct errors in marking individual layers.

Moreover, the detection and differentiation of motion artifacts can be challenging, particularly for individuals with limited OCTA experience. Various scoring systems have been proposed to evaluate the severity of motion artifacts, distinguishing between minor artifacts that do not disrupt the retinal vasculature and major artifacts that can significantly affect the imaging of retinal vessels. Therefore, being mindful of these factors is crucial for obtaining accurate and reliable OCTA results [39].

PRS is of interest to researchers in relation to many diseases, and, due to the polygenic basis of many diseases, it is used as an important indicator in numerous scientific studies in many specializations [36,40,41,42]. PRS in the group presented is described in our previous publications, where the detailed genetic testing process is described [30]. Sekimitsu et al. conducted a rare study examining the correlation between retinal OCT images and PRS, with a focus on evaluating the risk of cognitive impairment [39]. The research employed a logistic regression model to evaluate the link between Alzheimer’s disease and Parkinson’s disease PRS and retinal layer thickness. The findings affirmed the connection between genetic predisposition to neurodegenerative diseases and retinal OCT measurements. Additionally, the study identified potential predictive biomarkers for cognitive decline, suggesting that incorporating retinal measurements enhances the predictive accuracy of Alzheimer’s and Parkinson’s disease.

## 5. Conclusions

Although the superficial vascular network is not involved in AMD directly, it can be significantly altered by the change of shape of the retina above MNV. OCTA is used most reliably to assess the superficial weave, and, to a slightly lesser extent, a deep weave. It was found that both are independent of the genetic changes related to AMD. However, we expected that the characteristics of the above weaves may change secondary to external retina and RPE disorders, especially in the form of neovascularization. It cannot be ruled out that analysis based on a larger group would show such changes. We take into account that the need to exclude a large group of patients is a limiting factor, because the scale of vascular lesions in this group is probably the largest, but we do not currently have the capability of checking the vascular network measurements in this group.

## Figures and Tables

**Table 1:** Clinical characteristics of patients with AMD and control group—mean (standard deviation).

	AMD	Control	*p*-Value
N	89	43	∼
Age [years]	72.47 (7.99)	71.00 (7.04)	0.307
Sex [male/female]	40/49	10/33	0.026
Visual acuity [logMAR]	0.48 (0.45)	0.11 (0.18)	<0.001
Choroidal thickness [μm]	237.23 (105.45)	273.49 (82.03)	0.001

**Table 2:** Comparison of OCTA features values between group of patients with AMD and control group.

		AMD		Control		
Feature		M	SD	M	SD	*p*-Value
Diameter [μm]	Mean	47.97	7.10	46.67	1.47	0.4858
	Median	48.64	6.87	47.42	1.64	0.9512
	Variance	35.31	76.69	25.21	4.23	**0.0193**
	Skewness	−0.21	0.64	−0.38	0.44	**0.0457**
	Kurtosis	2.69	1.85	2.51	0.94	0.1231
Length [mm]	Mean	774.44	79.50	770.44	46.24	0.5359
	Median	578.41	76.37	603.62	63.09	0.0920
	Total	50.18	4.87	50.46	3.99	0.9729
	Variance	515,754.41	195,925.48	449,083.87	106,514.09	0.0521
	Skewness	1.97	0.55	1.85	0.48	0.2512
	Kurtosis	8.04	3.26	7.62	2.90	0.5247
Tortuosity	Mean	0.1139	0.0121	0.1155	0.0106	0.3614
	Variance	0.0190	0.0094	0.0181	0.0073	0.8270
	Skewness	3.04	0.72	3.12	0.80	0.8631
	Kurtosis	14.219	5.671	15.267	7.002	0.6392
Fractal Dimension	Mean	1.42	0.03	1.40	0.01	**0.0024**
	Variance	0.525	0.024	0.516	0.005	**0.0123**
Nodes		43.76	9.19	42.64	6.13	0.4683
VLD [%]		2.676	0.233	2.628	0.208	0.2574
VAD [%]		15.853	4.143	15.081	1.406	0.1373
BD [Nodes/mm]		0.863	0.129	0.842	0.079	0.3289
FAZ_*SCP*_ [mm^2^]		0.4737	0.3828	0.3446	0.0015	0.0616
FAZ_*DCP*_ [mm^2^]		0.5146	0.4261	0.4034	0.1555	0.6727

VLD—vessel length density; VAD—vessel area density; BD—branchpoint density; FAZ—foveal avascular zone; SCP—superficial capillary plexus; DCP—deep capillary plexus. Bold indicates statistically significant differences.

**Table 3:** Correlation and multivariate regression analysis result between the subjects’ characteristics and retinal morphological features with AMD PRS.

		Univariate		Multivariate (GEE)	
Feature		Pearson’s r	* p * -Value	Coefficient	* p * -Value
Covariants	AMD	∼	∼	0.8041	**<0.001**
	Age	∼	∼	−0.0013	0.915
	Sex	∼	∼	0.3924	**0.014**
	PED	∼	∼	0.3196	0.101
	MNV	∼	∼	0.0255	0.893
Diameter [μm]	Mean	0.042	0.630	1.344	0.179
	Median	0.040	0.650	−1.234	0.217
	Variance	0.016	0.853	−0.384	0.701
	Skewness	−0.049	0.574	−0.055	0.956
	Kurtosis	−0.100	0.253	−0.298	0.765
Length [mm]	Mean	−0.012	0.893	−0.709	0.479
	Median	−0.078	0.372	0.345	0.730
	Total	−0.124	0.157	−0.308	0.758
	Variance	0.070	0.422	−0.431	0.667
	Skewness	0.093	0.288	1.831	0.067
	Kurtosis	0.056	0.525	−1.517	0.129
Tortuosity	Mean	0.075	0.396	1.315	0.188
	Variance	0.043	0.621	−0.008	0.994
	Skewness	−0.142	0.105	−1.542	0.123
	Kurtosis	−0.144	0.101	1.157	0.247
Fractal Dimension	Mean	0.027	0.755	−0.508	0.611
	Variance	0.072	0.414	−0.078	0.938
Nodes		−0.027	0.756	0.430	0.667
VLD [%]		−0.098	0.263	0.124	0.901
VAD [%]		−0.011	0.899	0.059	0.953
BD [Nodes/mm]		0.024	0.788	−0.296	0.768
FAZ_*SCP*_ [mm^2^]		0.105	0.230	−0.133	0.894
FAZ_*DCP*_ [mm^2^]		0.103	0.241	0.410	0.682
MNV_*CC*_ area [mm^2^]		−0.054	0.692	−0.771	0.441
MNV_*OR*_ area [mm^2^]		0.104	0.692	1.398	0.162

VLD—vessel length density; VAD—vessel area density; BD—branchpoint density; FAZ—foveal avascular zone; SCP—superficial capillary plexus; DCP—deep capillary plexus; MNV—macular neovascularization; CC—choriocapillaris; OR—outer retina. Bold indicates statistically significant coefficients.

## Data Availability

The datasets generated and analyzed during the current study are available from the corresponding author on reasonable request.
